# Two Step Selection for Bias in *β* Chain V-J Pairing

**DOI:** 10.3389/fimmu.2022.906217

**Published:** 2022-07-14

**Authors:** Reut Levi, Yoram Louzoun

**Affiliations:** Department of Mathematics, Bar Ilan University, Ramat Gan, Israel

**Keywords:** TCR repertoire, TCR beta chain CDR3 repertoire, V-D-J rearrangement, junction length, selection

## Abstract

The *β* chain rearrangement in T cells is a two-step process where first *D_β_* and *J_β_* bind, and only then *V_β_* is joined to the complex. We here show that the frequency of human and mouse *V_β_ J_β_* combinations deviates from the one expected based on each gene usage frequency. This bias is observed mainly in functional (F) rearrangements, but also slightly in non-functional (NF) rearrangements. Preferred *V_β_ J_β_* combinations in F clones are shared between donors and samples, suggesting a common structural mechanism for these biases in addition to any host-specific antigen-induced peripheral selection. The sharing holds even in clones with J*_β_* 1 that share the same *D_β_* 1 gene. *V_β_ J_β_* usage is correlated with the Molecular Weight and Isoelectric Point in F clones. The pairing is also observed in the Double Positive cells in mice thymocytes, suggesting that the selection leading to such a pairing occurs before thymic selection. These results suggest an additional structural checkpoint in the beta chain development prior to thymic selection during the T cell receptor expression. Understanding this structural selection is important for the distinction between normal and aberrant T cell development, and crucial for the design of engineered TCRs.

## Introduction

T cells recognize self and foreign peptides through the interaction of their T-cell receptors (TCRs) with MHC bound peptides (1, 2). The TCR is located on the cell’s surface. Each host can have millions ofT cell clones with different TCRs (3). TCRs differ by their complementary determining region 3 (CDR3) sequence and the V, (D) and J alleles of their *α* and *β* chains. The TCR repertoire diversity is generated by two main mechanisms: the rearrangement of V (D) and J gene segments and by the nucleotide addition and removal at the junction between those segments (4, 5).

The TCR repertoire is then shaped through the T cell development. T cells arise from hematopoietic stem cells that migrate to the thymus where they require signals from nonhematopoietic stromal cells, such as thymic epithelial cells (TECs) and mesenchymal fibroblasts for survival, leading to positive selection based on ligand specificity (6, 7). A thymocyte whose TCR engages intra-thymic ligands and transduces intracellular signals can survive and undergo differentiation, while a thymocyte that is not signaled by its TCR undergoes death by neglect (8). This selection leads to two major lineages of T cells: CD4 T cells that recognize peptide antigens complexed to class II major histocompatibility complex (MHC) and possess helper functions, and CD8 T cells that recognize peptides complexed to class I MHC molecules and possess cytotoxic functions (9). In parallel, T cells are tested for reactivity with self-antigens to ensure that only those cells expressing acceptable antigen receptors (T cell receptors) mature.

The TCR *α* chain is composed of two segments (V*α* (variable) and *J_α_* (joining)). The *β* chain contains a third intermediate gene - *D_β_* (diversity). During the *β* chain rearrangement, there is first a recombination of one *D_β_* and one *J_β_*, followed by a recombination of *D_β_ J_β_* with *V_β_*, to form a rearranged *V_β_ D_β_ J_β_* gene segment.

In the *α* chain rearrangement, all the genes between the rearranged *V_α_* and *J*_α_ are removed. However, *V_α_* genes that are 5’ and *J*_α_ genes that are 3’ to the rearranged *V_α_* - *J*_α_ are still present allowing for multiple rounds of rearrangement (a process called editing). This process and the parallel process in the B cell Light Chain (LC) have been shown to induce a correlation between *V_α_* and *J*_α_ gene usage (10, 11).

**Figure 1:**
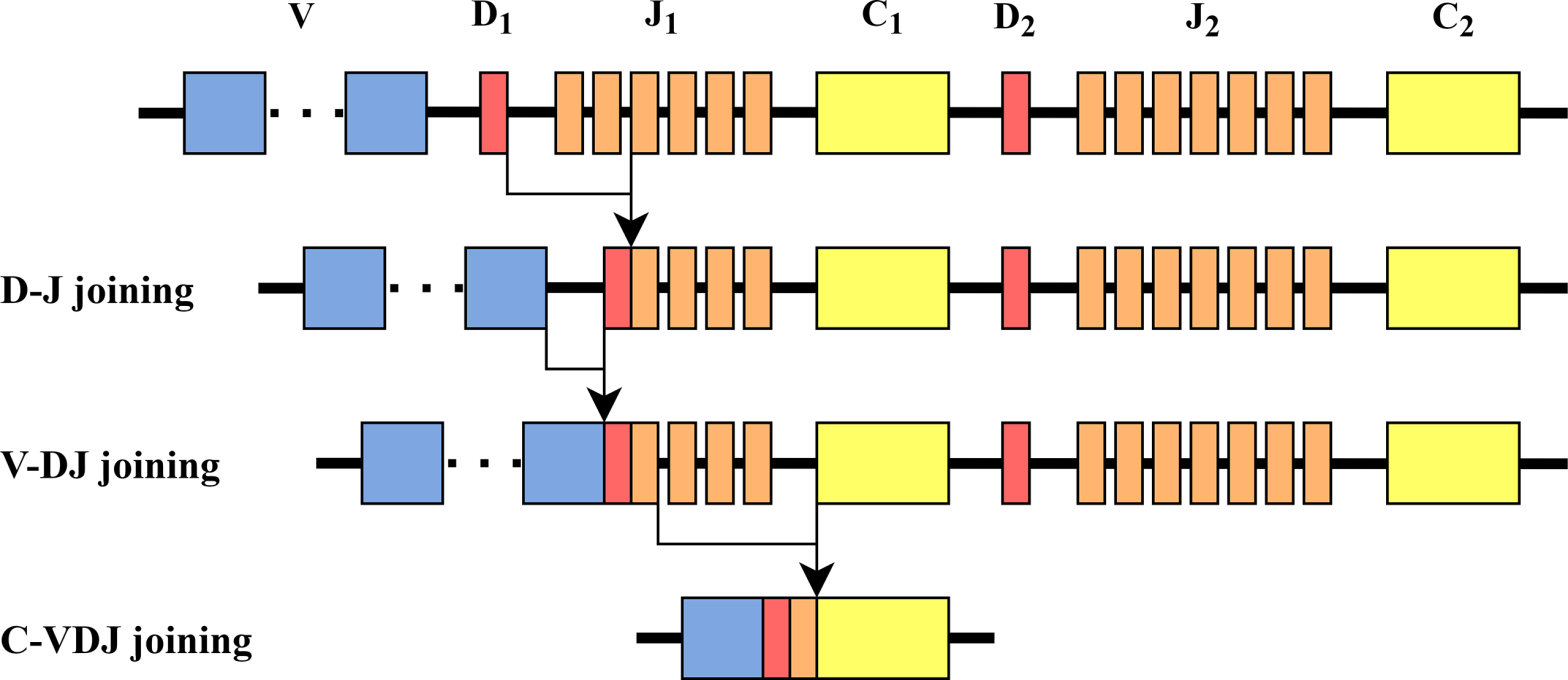
TCR *β* chain rearrangement. First, *D_β_* and *J_β_* are bound, then *V_β_* is bound to *D_β_ J_β_*, and then *V_β_ D_β_ J_β_* is bound to *C_β_*. The structure of the *β* chain in mice is similar to the one in humans.

In the *β* chain, there are two *D_β_* genes, *D_β_* 1 and *D_β_* 2 with very similar nucleotide sequences. *D_β_* regions can only recombine with downstream *J_β_* region elements. Thus, while *D_β_* 1 can recombine with elements from both the *J_β_* 1 and the *J_β_* 2 cassettes, *D_β_* 2 can only recombine with *J_β_* 2 genes (12) (see **Figure 1** for illustration). Thus, in TCRs that express *J_β_* 1 genes no editing could have happened. In principle, editing could happen once in a *J_β_* 2 gene, if the previous rearrangement was with a *J_β_* 1 gene.

The *D_β_* - *J_β_* rearrangement was reported to be biased, since *D_β_* and *J_β_* cassettes are joined (13, 14). However, at least in the *j_β_* 1 cassette, we expect *V_β_* - *J_β_* usage to be precisely the one expected from their individual frequencies. We here show that this is not the case. We show in human donors and mice a clear bias towards specific *V_β_ J_β_* combinations that are more frequent than expected from the *V_β_* and *J_β_* probabilities in both CD4 and CD8 T cells.

Specific *V* and *J* (in either *α* or *β* chains) were shown to differ between diseases (15, 16). A study from 2017 provides evidence for TCR expansion of clonotypes in autoreactive CD8+ T cells associated with type 1 diabetes. The authors found for example that the TRAJ53 (T cell Receptor Alpha) and TRAV29 pair were expressed in 31% of the clonotypes (17). Another example is the genetic predisposition to carbamazepine-induced Stevens-Johnson syndrome (SJS), a severe cutaneous hypersensitivity with high mortality (18). Another study found that the usage of *V*20 - 01 and *J*02 - 02 was increased in the *V_β_* - *J_β_* repertoire (*p* < 0.001) of the healthy volunteers compared to patients (19). Within a given host, the prevalence of specific gene segments and combinations of gene segments differ drastically. The variability in V and J gene usage is introduced before thymic selection (20), and are further shaped by epitope specificity in the periphery (21).

Multiple models were developed for the generation probability of TCRs and BCRs (22). Those include for example IGoR (23), which used out-of-frame receptor sequences to estimate rearrangement probability. To the best of our knowledge, all such models e.g (22, 24) treat *V_β_* and *J_β_* as independent, and approximate *P*(*V_β_*, *J_β_*) = *P*(*V_β_*) *P*(*J_β_*).

To summarise, specific V and J genes as well as V-J combinations have been shown to be more frequent than others in many contexts. We here show that the *V_β_* - *J_β_* usage is not only highly variable, but also differs from what is expected from their separate frequencies. We propose multiple measure that suggest that this pairing is the result of a structural selection step preceding thymic selection.

## Methods

### Notation

We used the notation presented in **Table 1** throughout the analysis.

**Table 1:** Notation.

*V_β_*	V gene in TCR
*J_β_*	J gene in TCR
*P* (*V_β_*)	The probability that a *V_β_* gene appears in a sample
*P* (*J_β_*)	The probability that a *J_β_* gene appears in a sample
*P* (*V_β_*, *J_β_*)	The probability that a (*V, J*) pair appears in a sample
*M* (*V_β_*, *J_β_*)	*P* (*V_β_*, *J_β_*) - *P* (*V_β_*) *P*(*J*_β_)
*C*(*i*,*j*)	Correlation between *M_i_* (*V_β_*, *J_β_*) and *M_j_* (*V_β_*, *J_β_*) of samples *i* and *j* over all gene combinations

### Study Subjects

We used four datasets in this analysis:

- **The RH dataset.** T cell receptor sequence data of alopecia patients before and during sensitisation with diphenylcyclopropenone and healthy volunteers at equivalent time points. The data contains 98 samples of *β* chains from 34 different patients. Participants were recruited from patients who had been diagnosed with alopecia, were aged between 18 and 70, identified as suitable for DPC treatment by a consultant dermatologist, and were now attending their first visit to the Alopecia Clinic at Salford Royal Hospital for DPC therapy. Twenty-nine of the individuals who participated in the study provided blood samples for TCR sequencing (TCRseq), for between one and four of the study time points (pre-sensitization, and at 2, 6, and 24 weeks of DPC treatment). Flow cytometry data was obtained for peripheral blood mononuclear cells (PBMCs) from 10 treated patients, and patch test data for 24 patients (25).
- **The MM dataset.** This dataset contains DNA sequences of T cells that were gathered and isolated from human tissues following organ donation, including blood, multiple lymphoid sites (bone marrow (BM), lymph nodes (LN), spleen (Spl)), and lungs. Donors were free of cancer and negative for hepatitis B, C, and HIV. The dataset contains four CD4+ and CD8+ T cell types: TCM (CD45RA- CCR7+), TEM (CD45RA- CCR7-CD69-), TRM (CD45RA- CCR7- CD69+), and TEMRA (CD45RA+ CCR7-) cells. (See Miron et al (26) for details.)
- **The Emerson dataset.** The Emerson dataset contains 786 immune repertoires (27). Each repertoire contains between 4,371 and 973,081 (avg. 299,319) distinct TCR sequences with a CDR3 length of 1 to 27 (avg. 14.5) amino acids. Each TCR is associated in each host with *V_β_* and *J_β_* genes and with a frequency. 340 repertoires are labeled CMV+, 421 are labeled CMV-, and 25 are of unknown status.
- **The LV dataset.** This dataset includes TCR sequences which were extracted from different T cell populations in mice, and then sorted for *γδ* TCR-/TCR*β*+, and CD44-/CD62L+. The data contains details of both TCR*α* and TCR*β* chains that were sequenced from more than 30 mice of different genetic backgrounds using adjusted sequencers. Pre-selection unsignaled Double Positive (DP) thymocytes were sorted based on CD4+, CD8*α*+, and CD69- gates from B6 or MHC-Knock Out animals (8).

For more details on each of the datasets see **Table 2**.

**Table 2:** Details for each of the datasets.

	Number of samples	Number of TCRs	Number of patients/mice	Number of reads
RH dataset	98	9,441,470	34	12,970,511
MM dataset	520	1,868,107	12	84,425,980
Emerson dataset	786	235,800,000	786	393,004,062
LV dataset	26	9,511,348	26	75,835,926

### Association Measure Between *V_β_* and *J_β_*

We compared for each sample the observed relative frequency of all (*V_β_ J_β_*) pairs *P*(*V_β_ J_β_*) and the expected frequency, defined as the product of the relative frequencies of *V_β_* and *J_β_*, *P* (*V_β_*) *P*(*J_β_*), and computed:


(1) $$M\left(V_{\beta },J_{\beta }\right)=P\left(V_{\beta },J_{\beta }\right)-P\left(V_{\beta }\right)P\left(J_{\beta }\right).$$

The probabilities are defined per sample (i.e. using only clones in this sample), and each clone was counted once, irrespective of the clone size. When we analyzed the *M* (*V_β_ J_β_*) distribution, we multiplied all the values by 100 to obtain values in percentages. Only *V_β_* and *J_β_* in the sample were considered.

### Correlation Between Samples

To quantify the similarity of deviation from a random pairing between samples, we computed the Spearman correlation between the *M* (*V_β_ J_β_*) values for all sample pairs.

Given a pair of samples *i* and *j*. Each sample contains only a subset of the *V_β_* and *J_β_* genes *V_β_ i_k_*, *J_β_ i_k_*, *V_β_ j_k_*, *J_β_ j_k_*. For each pair of samples, the common (*V_β_*, *J_β_*) pairs were taken s.t.


(2) $$S=\{\left(V_{\beta },J_{\beta }\right)|V_{\beta }\in V_{\beta }i_{k}\wedge V_{\beta }\in V_{\beta }j_{k}\wedge J_{\beta }\in J_{\beta }i_{k}\wedge J_{\beta }\in J_{\beta }j_{k}\}$$

We computed *M_i_* (*V_β_*, *J_β_*) and *M_j_* (*V_β_*, *J_β_*) for each pair in *S*, and computed the Spearman correlation for these pairs.


(3) $$C\left(i,j\right)=\rho_{Spearman}\left(M_{i}\left(V_{\beta },J_{\beta }\right),M_{j}\left(V_{\beta },J_{\beta }\right)\right)$$

### Detection of Anomalous *V_β_* - *J_β_* Pairs

To detect specific (*V_β_*, *J_β_*) pairs that deviate from the null model of random pairing, we computed for each pair in our dataset over all samples *P* (*V_β_*, *J_β_*) and *P*(*V_β_*) *P*(*J_β_*). Then, we performed a paired T-test on *P*(*V_β_*, *J_β_*) and *P*(*V_β_*) *P*(*J_β_*) for each pair separately. We applied a Benjamini-Hochberg correction (28) to the resulting probabilities. Significant pairs were defined as a corrected p-value less than 0.01.

### Null Models

We used two null models to compare our results. The first null model was generated by scrambling the *V_β_* and *J_β_* segments of the *V_β_ J_β_* pairs. Specifically, we used the clones in the sample and randomly reassigned the *V_β_* genes of the different clones, in each sample separately. When scrambling we scrambled at the clone level, and not at the read level (i.e. we did not scramble reads within a clone). The clone size or frequency was not used in the analysis. In addition, for the functional F clones, we also used the non-functional data (NF) clones as a comparison.

### Biochemical Features

For each dataset, we used only the F clones. We took all possible pairs of a given file, and for each pair, the total lengths of *V_β_* and *J_β_* was calculated. In addition, for each file and pair, we took its CDR3 and computed the sum of the Kyte Doolittle (KD), the Molecular Weight (MW) and the Isoelectric Point (IP) for all of its amino acids and averaged the values for each pair. Then, we calculated the *M* (*V_β_*, *J_β_*) values for each *V_β_*, *J_β_* pair in a given file, and averaged over all the pairs in the same dataset. We then computed the Spearman correlation between the sum of the gene lengths, the KD, the MW or the IP and the mean *M* (*V_β_*, *J_β_*) values.

### Statistical Analysis

- To test the correlation between different samples, only the common pairs of the two samples were taken. We calculated for each pair (*V_β_*, *J_β_*) the *M* (*V_β_*, *J_β_*) and *M*_1_ (*V_β_*, *J_β_*), where *M*_1_ is the measure for the mixed data both for the real data and for the first null model. Next, we calculated the *Spearman correlation coefficient* on these two samples.
- In order to test whether the distribution of *M* (*V_β_*, *J_β_*) on the real data is different from the distribution of *M* (*V_β_*, *J_β_*) on the null model, we performed the *two-sided Kolmogorov-Smirnov statistic on two samples* (29).
- To test whether the standard deviation of *M* (*V_β_*, *J_β_*) on the real data is different from the standard deviation of *M* (*V_β_*, *J_β_*) on the null model, we used a *two-sided T-test on two related samples of scores*. We also used this test to identify which pairs have a signal. For each pair (*V_β_*, *J_β_*), we calculated over all samples *P* (*V_β_*, *J_β_*) and *P* (*V_β_*) *P* (*J_β_*), and performed the above test for *P* (*V_β_*, *J_β_*) and *P* (*V_β_*) *P* (*J_β_*) for each pair separately. We applied the *Benjamini-Hochberg correction* (28).
- To test whether the correlation vector is significantly different, we calculated the *two-sided T-test for the mean of one group of scores*, where the expected value is 0. Moreover, in order to test whether the correlations within a patient are different from the correlations between different patients, we used a *two-sided T-test for the means of two independent samples of scores*.
- In order to determine how two factors impact a response variable, and to determine whether or not there is an interaction between the two factors on the response variable, we used a *two-way ANOVA test*.
- For analyzing the division between samples from different patients we used a *one-way chi-square test*. We defined neighbors as consecutive samples with the same compartment/sample. Note that this only shows the deviation from a random order.

## Results

### *V_β_*, *J_β_* Are Preferentially Strongly Paired in Functional Rearrangement, and at a Much Lower Level in Non-Functional Rearrangements

We used 4 datasets for the analysis, RH, MM, Emerson and the LV datasets (see Methods). Each dataset contains several patients (see Methods). Some contain samples from different compartments (CD4 vs CD8), as well as different conditions (healthy vs sick in different conditions). We analyzed only DNA sequencing based repertoire, and each sample contains both functional and non-functional clones. We removed all samples with less than 1000 clones. In the Emerson dataset, we used a random sample of 100 patients. We ignored the frequency of each clone in each donor to avoid biases induced by differential amplification. We grouped *V_β_* gene and *J_β_* gene representations into 2 fields gene notation (e.g., V01-02 and J01-02), and ignored allelic differences (V01-02:01 → V01-02).

**Figure 2:**
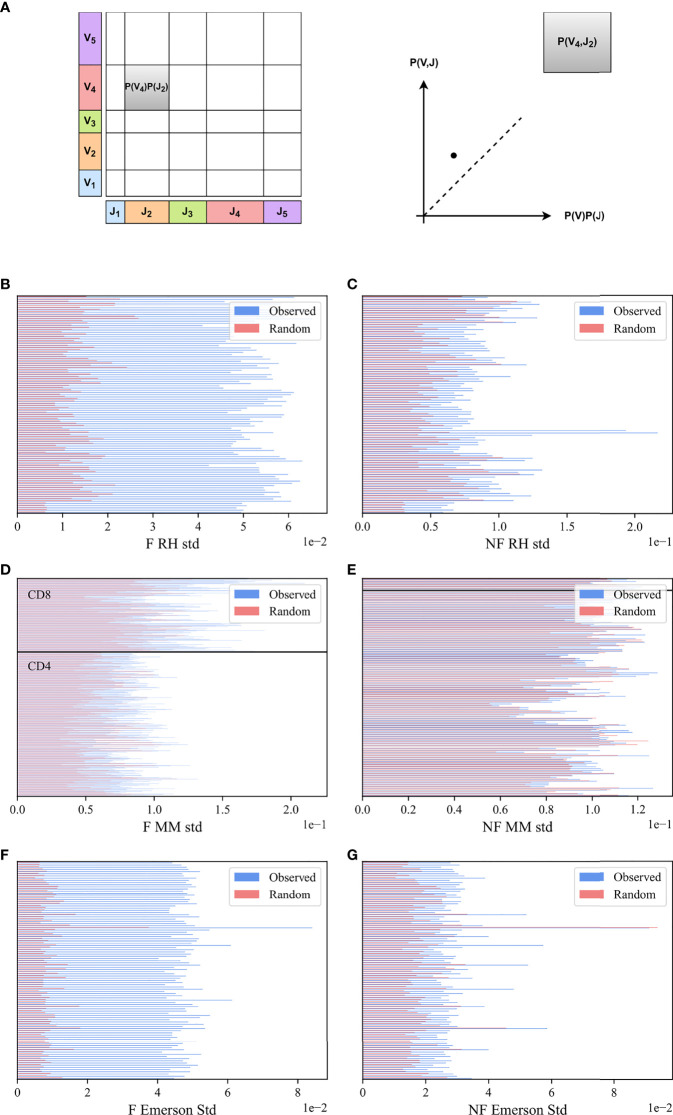
*M* (*V_β_*, *J_β_*) bias. **(A)** Schematic explanation *M* (*V_β_*, *J_β_*) measure. We computed the marginal frequency in a sample of *J_β_* and *V_β_* (X and Y axes), defined to be the fraction of clones using each. We then computed their product (size of rectangles), and compared this product with the actual number of clones that use a given *V_β_*, *J_β_* pair. **(B, C)** The standard deviation of *M* (*V_β_*, *J_β_*) values for the RH dataset. The blue bars describe the real F clones values **(B)** and the real NF clones values **(C)** while the pink bars represent the null model. **(D, E)** The standard deviation of *M* (*V_β_*, *J_β_*) values for the MM dataset, with the same colors. All samples above the black line are CD8 T-cells, and below are CD4 T-cells. **(F, G)** Same results for the Emerson dataset.

To test whether *V_β_* and *J_β_* usage frequencies are paired, we compared the *V_β_*, *J_β_* frequency distribution of functional (F) clones in each sample with the one expected under the null hypothesis of independent pairing. To compute that, the marginal probability of each *J_β_* (i.e., the probability that a randomly chosen clone would have a given *J_β_* - x-axis in **Figure 2A**, and the same for *V_β_* - y-axis in **Figure 2A**) must be computed. Their product is the expected *P* (*V_β_*) *P* (*J_β_*) value (rectangle area in **Figure 2A**). As a schematic example, for the pair (*V*_4_, *J*_2_) in **Figure 2A**, *P* (*V*_4_, *J*_2_) is larger (i.e., has more clones) than expected by *P* (*V*_4_) *P* (*J*_2_) (i.e., it is above the diagonal in the observed vs expected plot).

To systematically quantify this deviation, we computed for each (*V_β_*, *J_β_*) pair in a given sample:


(4) $$M\left(V_{\beta },J_{\beta }\right)=P\left(V_{\beta },J_{\beta }\right)-P\left(V_{\beta }\right)P\left(J_{\beta }\right)$$

While, in principle, the value of *M* (*V_β_*, *J_β_*) is expected to be zero for random pairing, finite size effect can induce deviations from zero. We thus compared the distribution of *M* (*V_β_*, *J_β_*) to the null model results, where the *V_β_* and *J_β_* of the clones were scrambled. Specifically, we used the clones in the sample and randomly reassigned the *V_β_* genes of the different clones (see Methods). The distribution of *M* (*V_β_*, *J_β_*) for the F clone is wider than for the null model, as further shown.

To quantify the difference, we computed the standard deviation in the real and null model of *M* (*V_β_*, *J_β_*) distributions and performed a paired T-test on the standard deviations in the real and null models over all samples for each dataset. The standard deviation of the real clones is larger than the null model for all files (**Figures 2B, D, F)** for RH, MM and Emerson datasets, p-value 8.3e-89, 2.04e-129 and 2.23e-99, respectively). We further performed a Kolmogorov-Smirnov test (29) on the distributions in the real data and the null model for all samples together, with a very significant difference (*p* – *value* < 1*e* – 100, 7.24*e* – 89,1*e* – 100 for the respective datasets).

In order to check whether the bias of *V_β_ J_β_* usage also exists in the NF clones, we computed *M* (*V_β_*, *J_β_*) in the non-functional data (for all the datasets), and found a deviation from the *V_β_* and *J_β_* null model also in NF rearrangements. The standard deviation of the NF clones is slightly larger than the standard deviation of the null model (p-value 1.79e-24, 5.26e-16 and 3.15e-9, respectively) (**Figures 2C, E, G**). The deviation from the null model in NF clones is much smaller than in F clones. A Kolmogorov-Smirnov test for all samples together shows a significant deviation from the null model for the NF clones for two out of the three datasets studied (p-value 1.45e-27, 0.14, 3.61e-50). To summarise, a very strong deviation from the null model is observed in F clones, and a weak yet significant deviation exists in NF clones.

### *V_β_*, *J_β_* Preferential Pairing Is Affected by T Cell Compartment or by Donor Condition

We further checked if there is a difference between the CD4 and the CD8 T-cells (**Figure 2D**, all the samples above the black line are CD8 T-cells, and below are CD4 T-cells) in the F clones. The standard deviations in the CD8 cells are larger than in the CD4 cells (CD8 mean std 0.12 vs CD4 mean std 0.08, p-value 6.89e-53 for the F clones). We tested whether there is a difference between the groups using a two-way ANOVA test (p-value of 4.89e-91 for CD4 vs CD8, 1.5e-71 of observed vs the null model, and a limited yet significant interaction effect). When combining F and NF, the two-way ANOVA test yields a p-value of 0.005 for CD4 vs CD8, 0.002 for Real NF vs Random, but as expected no interaction effect (p=0.98)

The difference between CD4 and CD8 T cells occurs during or after thymic selection and is antigen induced, suggesting at least a partial effect of antigen induced selection on pairing. However, the difference is small, suggesting that other more generic mechanism may drive this pairing.

We further tested in the RH dataset whether there is a difference between HV (healthy volunteers) samples and samples of patients who had been diagnosed with alopecia by using a two-way ANOVA test. We obtained a p-value of 3.74e-4 for healthy vs unhealthy, 1.62e-125 for observed vs random and 0.244 for the interaction effect, suggesting that the difference between the real data and the null model is not induced by the condition of the host (at least for alopecia).

To summarize, a bias *V_β_ J_β_* usage was found in both F and NF clones, with more significant differences for the F clones, and limited differences between CD4 and CD8 T cells. Antigen-driven selection may be a simple explanation for the differences we found between F and NF clones. An alternative model may be preferential pairing of *V_β_* and *J_β_* during rearrangement, or a structural selection preceding selection in the thymus. We here provide multiple lines of evidence for the last possibility, with a major contribution of structural selection.

### *V_β_*, *J_β_* Bias Is Not Mediated by the *D_β_* Gene Used

**Figure 3:**
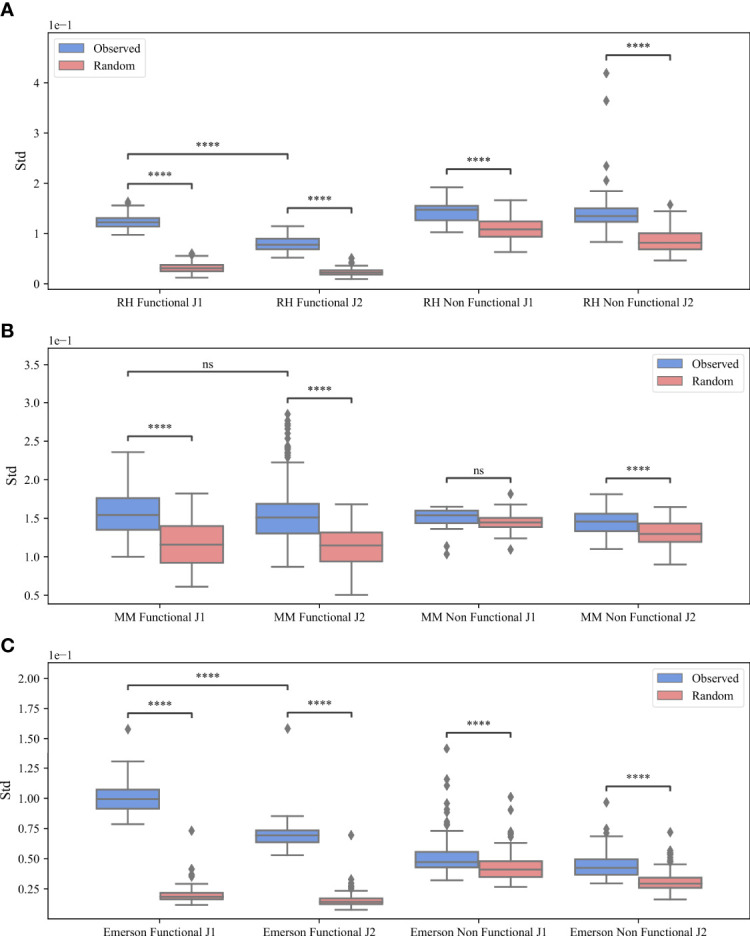
*M* (*V_β_*, *J_β_*) bias. The standard deviation of *M* (*V_β_*, *J_β_*) values for the RH dataset **(A)**, the MM dataset **(B)** and the Emerson dataset **(C)**. The x-axis represents whether the clones are functional or non-functional and the different *J_β_* gene families, while the y-axis represents the standard deviation. The blue boxes describe the real clones values and the pink boxes represent the randomly generated clones. The boxes extend from the Q1 to Q3 quartile values of the data, with a line at the median (Q2). The whiskers extend from the edges of the box to show the range of the data, they extend to 1.5 * IQR (IQR = Q3 - Q1) from the edges of the box, ending at the farthest data point within that interval. Outliers are plotted as separate dots. A T-test was performed to test how significant the differences between the observed and random standard deviation of the *M* (*V_β_*, *J_β_*) values are, where ****p-value < 0.0001, ***p-value < 0.001, **p-value < 0.01, *p-value < 0.05 and ns p-value > = 0.05.

Beyond the models above, a simple explanation for the preferential pairing could be that some *V_β_* prefer some *D_β_* 1 or *D_β_* 2, which in turn prefer some *J_β_*, leading to indirect pairing, between *V_β_* and *J_β_*. T-cells have only two *D_β_* genes and two cassettes of *J_β_*, where *j_β_* genes in the *J_β_* 1 cassette bind to *D_β_* 1 and *j_β_* genes in the *J_β_* 2 cassette can bind both _Db_ *D*_\β genes. Thus, if the preferential *V_β_ J_β_* binding would be induced by the choice of a specific *D_β_* gene, it should disappear, when only clones with *J_β_* genes in the *J_β_* 1 cassette are analyzed. We thus separated all clones according to their *J_β_* gene groups, and analyzed the pairing in each group separately (*J_β_* 1 and *J_β_* 2). Formally, we separated each repertoire into two sub-repertoires according to the family of the *J_β_* gene. Then, we calculated *M* (*V_β_*, *J_β_*) for each sub-repertoire, and repeated the tests above (**Figure 3**).

For all datasets, the standard deviation of the real data is larger than the standard deviation of the null model (blue vs pink boxes in **Figure 3**). The difference between the real data and the null model is larger for the *J_β_* 1 gene than in the *J_β_* 2 (**Figures 3A** vs **3B**, but there is still a difference in the *J_β_* 2 as well. The mean standard deviation for the F clones is 0.12 (*J_β_* 1) vs 0.07 (*J_β_* 2) for RH, 0.156 (*J_β_* 1) vs 0.153 (*J_β_* 2) for MM and 0.099 (*J_β_* 1) vs 0.069 (*J_β_* 2) for the Emerson data. We performed a T-test for the F clones between *J_β_* 1 and *J_β_* 2 and found that for the RH and Emerson datasets there are significant differences between *J_β_* 1 and *J_β_* 2 (*p* < 7.04*e* - 57 and *p* < 1.92*e* - 44) compared to MM where we found no significant difference (0.25). In addition, for all datasets, the difference between the real data and the null model is significant for both *J_β_* 1 and *J_β_* 2 genes (*p* < 0.0001 forall comparisons), except for the NF clones in the *J_β_* 2 MM dataset (*p* > 0.05). Thus, selection for *D_β_* cannot explain the observed bias.

### *V_β_*, *J_β_* Preferential Pairing Is Correlated Between Patients and Within Compartments Between Patients, for Both F and NF Clones

**Figure 4:**
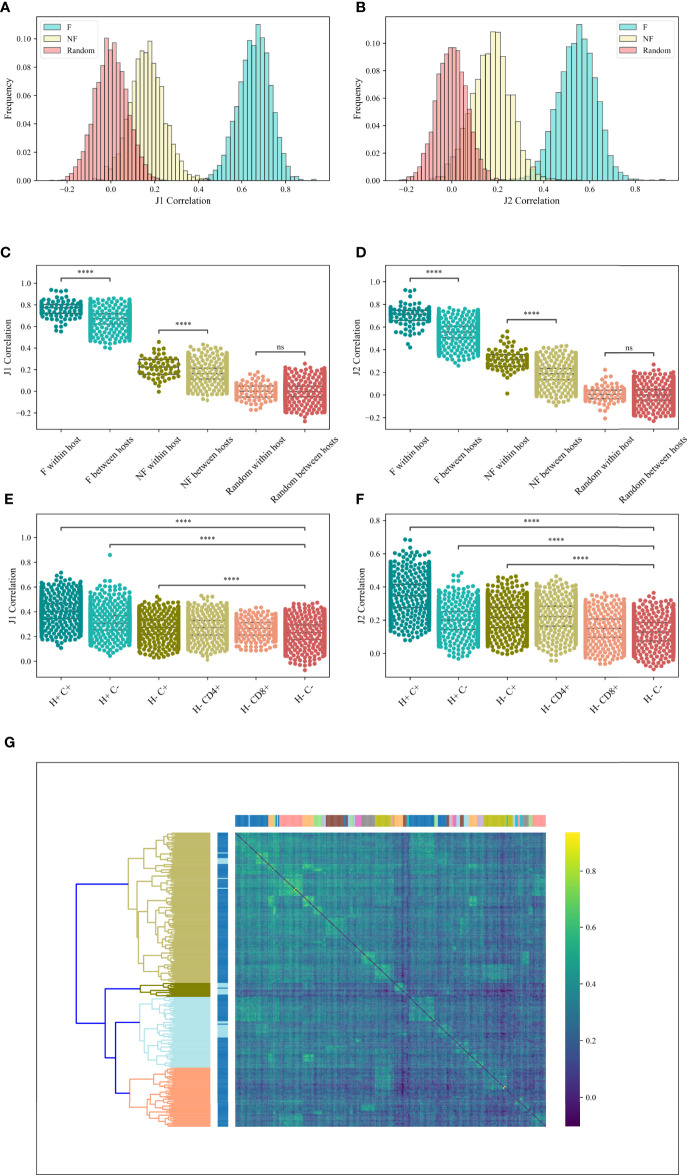
*M* (*V_β_*, *J_β_*) correlation. **(A, B)** The correlations histogram of the *M* (*V_β_*, *J_β_*) values for the *J_β_* 1 family gene **(A)** and the *J_β_* 2 family gene **(B)** in the RH dataset. The blue histogram represents the F clones, the beige histogram is the NF clones and the pink histogram represents the null model. **(C, D)** Correlations of *M* (*V_β_*, *J_β_*) values for the *J_β_* 1 family gene **(C)** and the *J_β_* 2 family gene **(D)** in the RH dataset within host and between hosts for F, NF and random clones. Star symbols follow the previous plot. **(E, F)** Correlations of *M* (*V_β_*, *J_β_*) values for the *J_β_* 1 family gene **(E)** and the *J_β_* 2 family gene **(F)** in the MM dataset, where H+ represents within host, H- represents between hosts, C+ represents within compartment, C- represents between compartments. **(G)** Heatmap of the correlations of *M* (*V_β_*, *J_β_*) values for the *M J_β_* 1 family gene of the F clones in the MM data set. At the top we colored according to a patient, while on the left we colored according to the compartments (CD4 or CD8).

If pairing is induced by structural selection, it should be similar between hosts and samples. Alternatively, if the pairing is antigen-driven, we would expect it to differ between hosts and especially between CD4 and CD8 T cells. To test for that, we computed the Spearman correlation between the *M* (*V_β_*, *J_β_*) values for all sample pairs from different hosts in the RH dataset, and computed the distribution of the correlation (**Figures 4A, B**) for the F clones (blue bars), the NF clones (brown bars) and the null model (beige bars). For each pair of samples, only the common (*V_β_*, *J_β_*) pairs of these two samples were taken. One can clearly see that the correlation of the null model is centered around zero, while the correlation of the real data is centered around 0.6 for the F clones and 0.2 for the NF clones. The same holds for the other datasets (data not shown). The similarity between samples is consistent in the different *J_β_* gene families (*J_β_* 1 - **Figure 4A** and *J_β_* 2 - **Figure 4B**). Interestingly, even for the NF clones, the correlation is centered around positive values, albeit lower than the F clones, suggesting a genetic mechanism in addition to the structural one (ANOVA test, *p* < 1*e* - 100 for both *J_β_* 1 and *J_β_* 2). T-tests between the F and NF correlation distribution and the random distribution *p* < 1*e* - 100 for both *J_β_* 1 and *J_β_* 2).

Samples from the same host share the same genetic *V_β_* and *J_β_* loci compositions. If the *V_β_*, *J_β_* pairing is affected by a genetic bias, we expect samples from the same host to have more similar biases than between hosts. Indeed, a slightly higher correlation was observed in samples within-host than between hosts for both F and NF samples (T-test between the correlations within-host and the correlations between hosts for both *J_β_* 1 and *J_β_* 2 genes: F clones – *p* < 6.68*e* - 39 (*J_β_* 1) and *p* < 3.52*e* - 75 (*J_β_* 2), NF clones – *p* < 3.37*e* - 8 (*J_β_* 1) and *p* < 3.39*e* - 50 (*J_β_* 2), null model – *p* = 0.91 (*J_β_* 1) and *p* = 0.22 (*J_β_* 2) (**Figures 4C, D**, RH dataset).

In contrast, CD4 and CD8 T cells recognize completely different epitopes (presented by either MHC class I or class II). If antigen-driven selection also contributes to the paring mechanisms, we would expect CD4 T cells clones to have more similar pairing to other CD4 T cells than to CD8 T cells and vice versa.

To test for that, we explored the correlations between the values of *M* (*V_β_*, *J_β_*) among patients and compartments and compared the effect of compartment vs the effect of hosts. We analyzed the F clones of the MM dataset. In order to check whether samples of the same patient or the same compartment are more similar, we computed the correlation within hosts (H+), within compartments (C+) and between hosts (H-) and between compartments (C-). For *J_β_* 1 family, the correlations within compartment (H-C+) were only slightly higher than the one between compartments (H-C-) (0.27 vs 0.23 on average, T-test *p* < 2.46*e* - 225). However, the correlations within hosts (H+C-) were much higher (0.31 on average, T-test vs H-C- *p* <1.23*e* - 148). The correlations within-host and compartment (H+C+) were the highest (0.4 on average, T-test vs H-C- *p* < 1*e* - 100) (**Figure 4E**). For *J_β_* 2 family gene, the correlations within compartment (H-C+) and within hosts (H+C-) were slightly higher than the one between compartments (H-C-) (0.2 vs 0.12 on average, T-test *p* < 1*e* - 100). The correlations within-host and compartment (H+C+) were the highest (0.34 on average, T-test vs H-C- *p* < 1*e* - 100) (**Figure 4F**). In addition, two-way ANOVA was done for the *J_β_* 1 and the *J_β_* 2 family genes (*p* < 0.0001 and *p* < 1.6*e* - 35 for CD4 vs CD8, *p* < 1*e* - 100 for functional vs random for both families, and *p* < 0.0046 and *p* < 1.97*e* - 63 for the interaction effects, respectively).

We further tested if samples in the same host or same compartment were clustered together, we clustered the correlations using hierarchical clustering based on the Euclidean distance and complete linkage [**Figure 4E** - Top coloring is according to a patient, and left coloring is according to the compartments (CD4 or CD8)]. As one can see, samples from the same patient (neighbors of the same color on the top) are grouped together and there is a clean division between samples from different patients, with no separation between CD4 and CD8 T cells (chi-square test vs random label permutations *p* < 2.39*e* - 124). Compartments were not grouped more than expected randomly. The stronger similarity within donors further suggests a stronger effect of the genetically induced bias in the rearrangement mechanism than the antigen-driven one, and the correlation in all samples that is higher in the F than NF clones suggest an important component of structural selection.

### Biased Pairs Are Consistent Among Different Datasets and Among Compartments

If the selection is indeed genetic/structural and it happens before any antigen-induced selection, we expect the pairs selected for and against to be consistent among datasets, and between CD4 and CD8 T cells. To test that, we analyzed all the (*V_β_*, *J_β_*) pairs with the most significant deviation from random pairing (*p* < 0.01), and found that there is a large overlap in these specific pairs between the different datasets (107 vs 49.66 expected randomly, chi-square *p* < 4.1*e* - 16 for the *J_β_* 1 family, and 82 vs 45.9 expected randomly, chi-square *p* < 9.9*e* – 8 for the *J_β_* 2 family). In addition, most of the significant pairs that overlap between the three datasets have the same deviation sign (98/107, i.e., 91% for *J_β_* 1 and 70/82, i.e. 85% for *J_β_* 2).

**Figure 5:**
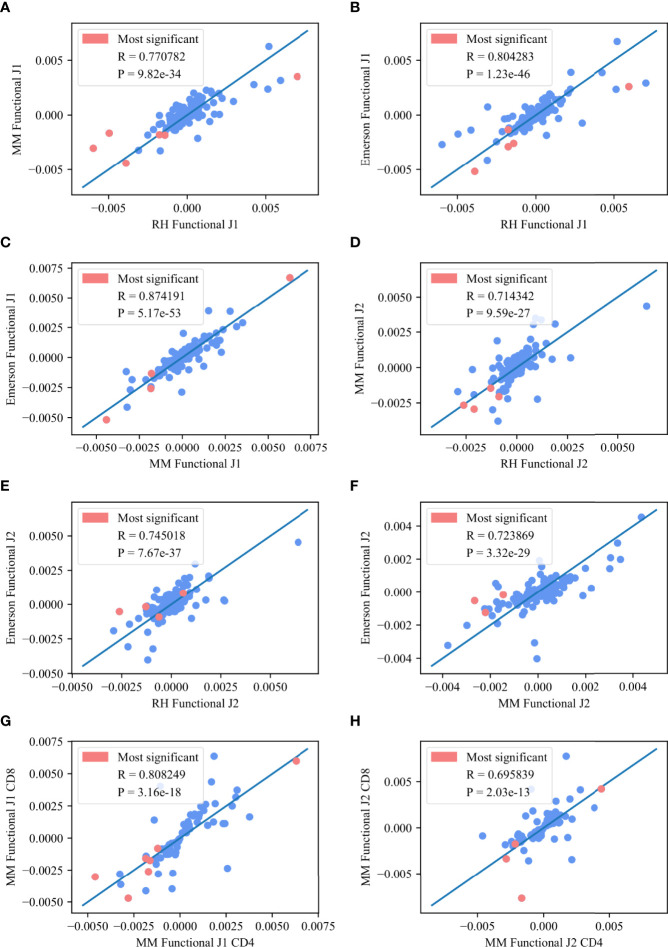
Deviation from random pairing. **(A–F)** *M* (*V_β_*, *J_β_*) values between any two functional datasets for the *J_β_* 1 family gene **(A–C)** and the *J_β_* 2 family gene **(D–F)**. The pink points represent the common pairs of the 10 most significant pairs between these two datasets. **(G, H)** *M* (*V_β_*, *J_β_*) values for the CD8 T-cells in the MM functional for the *M J_β_* 1 family gene **(G)** and the *J_β_* 2 family gene **(H)** asa function of *M* (*V_β_*, *J_β_*) values for the CD4 T-cells in the MM functional for the same family gene. The pink points represent the common pairs of the 10 most significant pairs between these two data sets.

We further analyzed the common significant pairs (*p* – *value* < 0.01) between any two datasets, and compared *M* (*V_β_*, *J_β_*) values among datasets, or among compartments in the same dataset (CD8 vs CD4 cells). Indeed, *M* (*V_β_*, *J_β_*) is highly consistent among the datasets for both *J_β_* 1 and *J_β_* 2, with a higher average correlation for *J_β_* 1 (0.81 vs 0.72) (**Figure 5**, where the pink points represent the common pairs of the 10 most significant pairs between each of the two datasets). The same happens between compartments (*J_β_* 1 R=0.8 correlation and *J_β_* 2 R=0.69, *p* < 3.16*e* – 18 and *p* < 2.03*e* - 13, respectively).

The top *V_β_* - *J_β_* preferential gene pairings were *V*05 – 01 / *J*01 - 05, *V*29 – 01 / *J*01 - 01, *V*05 – 04 / *J*01 - 05, *V*29 – 01 / *J*01 - 06, *V*06 – 01 / *J*01 - 05, *V*29 – 01 / *J*01 - 05, *V*09 – 01 / *J*01 - 05, *V*19 – 01 / *J*01 - 05 and *V*11 – 02 / *J*01 - 05 for the *J_β_* 1 family and *V*24 – 01 / *J*02 - 07, *V*20 – 01 / *J*02 - 06, *V*06 – 05 / *J*02 - 03, *V*07 – 07 / *J*02 - 05, *V*20 – 01 / *J*02 - 02, *V*11 – 03 / *J*02 - 07 and *V*07 – 07 / *J*02 - 07 for the *J_β_* 2 family. All of these pairs are in the 10 most significant pairs for each dataset and overlap between at least two of the three datasets. This high correlation again suggests a generic structural mechanism that is not antigen driven.

### Bias Exists in an Early Stage of Thymic Development Before Antigen Mediated Selection

**Figure 6:**
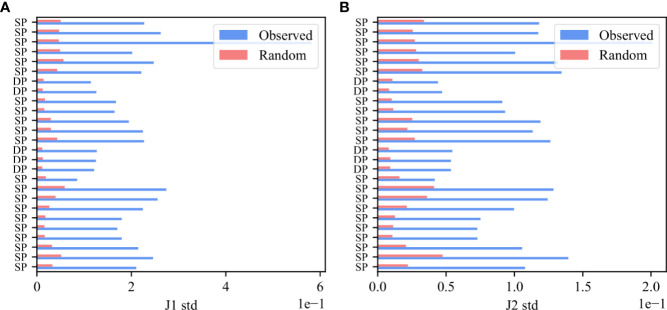
Bias in Double Positive samples in mice thymocytes. The standard deviation of *M* (*V_β_*, *J_β_*) values for the *J_β_* 1 family gene **(A)** and for the *J_β_* 2 family gene **(B)**. The blue bars describe the F clones values and the pink bars represent the null model.

To further show that the bias is due to rearrangement and structural selection, the *V_β_*,*J_β_* preferred pairing should be present even before any antigen-induced selection event, and should be much larger in F than NF clones even early in thymic development. To test for that, we analyzed mice thymocytes, and computed deviation from random pairing in samples of Double Positive cells. As was done for the human data, we computed for each functional sample the standard deviation of the real and null model $M(V_\β,J_\β)$ i.e. M(V_b_,J_b_) distributions and performed a paired T-test on the standard deviations in the real and null models (**Figure 6**). Indeed, there is a very clear bias already in the Double Positive samples both for the *J_β_* 1 family (**Figure 6A**, p-value 6.47e-11) and for the *J_β_* 2 family (**Figure 6B**, p-value 8.89e-13), and the preferred pairing is much stronger in F than NF.

### Gene Usage Is Associated With Junction Length

We have shown that (*V_β_*, *J_β_*) pairing exists even in the *J_β_* 1 family genes that only use *D_β_* 1 in NF clones. However, there is no direct rearrangement of *V_β_* and *J_β_*, so it is unclear how can such a pairing occur. We hypothesized that the pairing between *V_β_* and *J_β_* is through the length of the junction between *V_β_* and *D_β_* and the length of *J_β_* and *D_β_*. In other words, different *J_β_* genes favor different junction lengths and so do different *V_β_* genes. This length preference combined with a preference for intermediate length CDR3 genes (5) can induce an indirect pairing mechanism.

**Figure 7:**
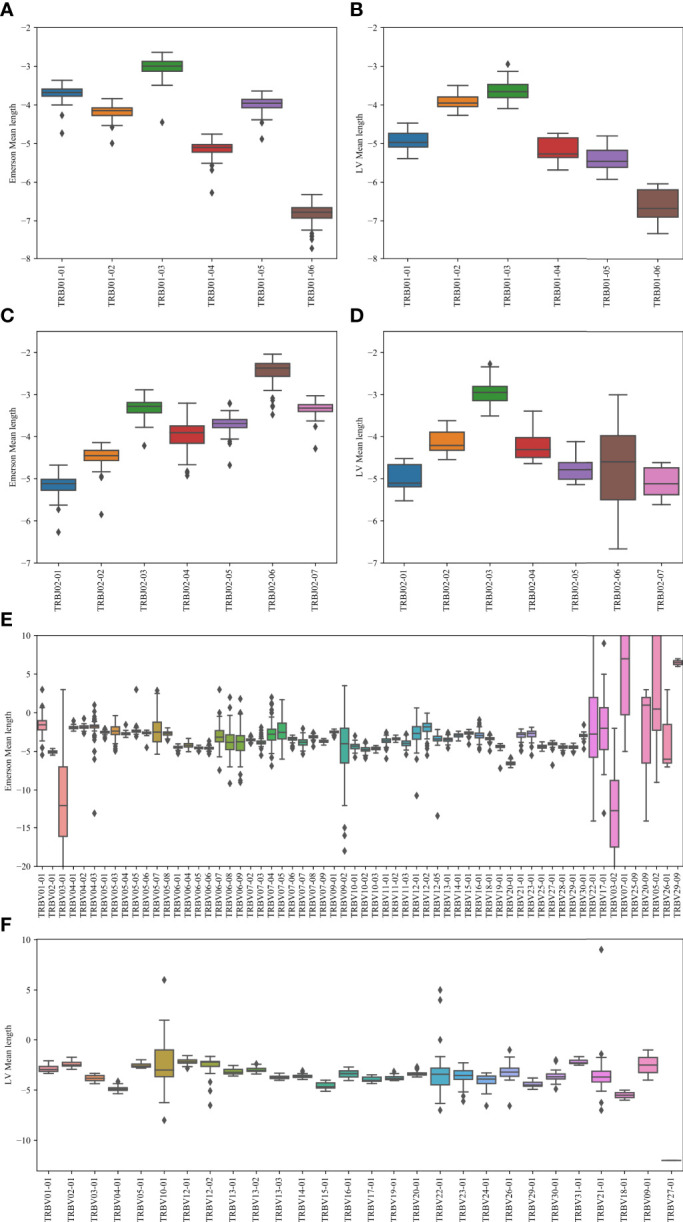
Junction length. In the *J_β_* plots, the junction length is the average distance between the beginning of the germline *J_β_* gene, and the end of the germline *D_β_* gene. In the *V_β_* plot, the difference is between the *V_β_* germline and the *D_β_* germline genes. **(A, B)** The mean distance for the Emerson dataset **(A)** and for the LV dataset **(B)**. The x-axis represents the various *J_β_* genes within the *J_β_* 1 family gene. **(C, D)** The mean distance values for the Emerson dataset **(C)** and for the LV dataset **(D)**. The x-axis represents the various *J_β_* genes within the *J_β_* 2 family gene. **(E, F)** The mean distance values for the Emerson dataset **(E)** and for the LV dataset **(F)**. The x-axis represents the various *V_β_* genes within the *J_β_* 1 family gene. The interpretation of the boxes follows the previous plots.

To test that, we calculated the average number of insertions minus the number of deletions for each *V_β_*, *D_β_* pair, and *D_β_*, *J_β_* (**Figure 7**). Specifically, we computed the junction lengths by inferring the initial and final position of the germline *D_β_* gene, and computing the final position of the germline *V_β_* gene and the initial position of the germline *J_β_* gene, based on the beginning and end of the variable region. The junction length (that can be negative) is the difference between the end of germline *V_β_* and the beginning of germline *D_β_* for the first junction and similarly with *D_β_* and *J_β_* for the second junction. For the *J_β_* gene, we performed this analysis on *J_β_* 1 and *J_β_* 2 separately (**Figures 7A, B**), while for the V gene, we performed it only on *J_β_* 1 (**Figure 7C**). The results are similar for *J_β_* 2 (data not shown). Indeed, consistently, *J*01 - 03 has the highest average junction length and *J*01 - 06 has the lowest for the *J_β_* 1 family gene. For the *J_β_* 2 family, *J*02 - 06 has the highest, and *J*02 - 01 has the lowest junction length. Similarly, different *V_β_* have different junction lengths. Thus, *V_β_* and *J_β_* may match to ensure the proper CDR3 length.

### *V_β_*, *J_β_* Pairing Is Associated With Biochemical Properties of Receptors

To test that pairings that produce intermediate receptor sizes are preferred, we computed for the receptor with each (*V_β_*, *J_β_*) pair in each F sample, the average length (the sum of *V_β_* and *J_β_* genes length in AA), molecular weight (MW), hydrophobicity (as measured by the kyte doolittle -KD score), and charge (as measured by the iso-electric point - IP).

**Figure 8:**
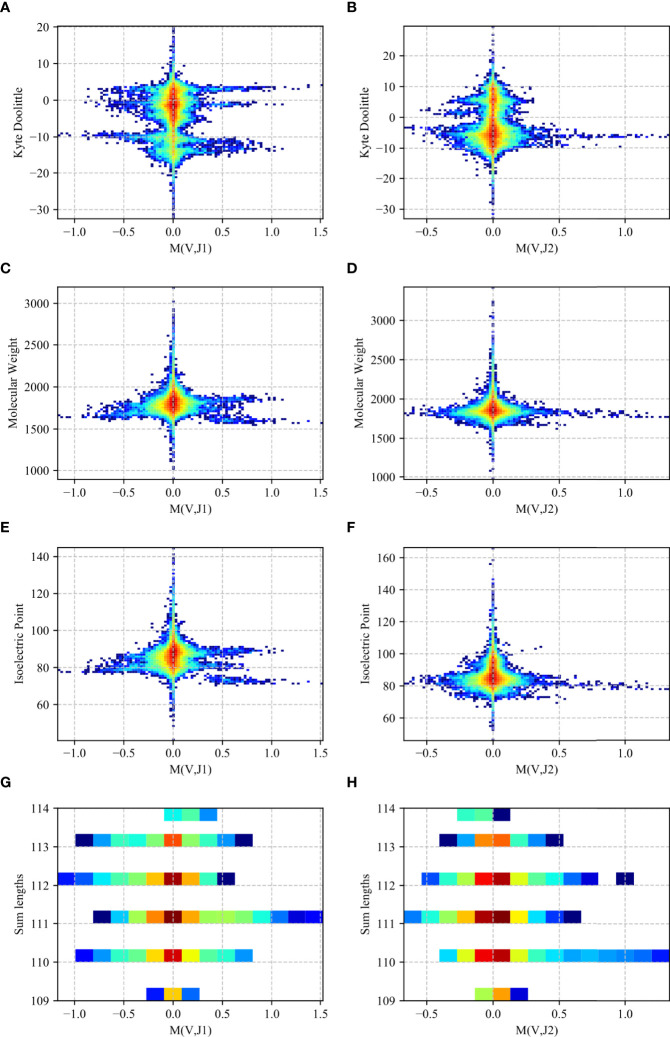
Two-dimensional histogram (RH dataset). 2D histogram where the x-axis represents the *M* (*V_β_*, *J_β_*) values for the *J_β_* 1 family gene **(A, C, E, G)** and the *J_β_* 2 family gene **(B, D, F, H)** while the y-axis represents the Kyte-Doolittle values **(A, B)**, Molecular Weight values **(C, D)**, Isoelectric Point values **(E, F)** and the sum of the gene lengths values **(G, H)**. The colors represent the fraction of clones with such a value. Blue colors are low frequencies, while red colors are high.

We computed a two-dimensional histogram on the RH dataset for both the *J_β_* 1 and *J_β_* 2 family genes for each measure (**Figure 8**). One can clearly see a preference of high *M* (*V_β_*, *J_β_*) values for intermediate to low isoelectric points, molecular weights and length, and a more complex picture for the KD. In other words, V and J genes pair to favor intermediate polarity and weight, but also some specific polarity of the resulting receptor.

## Discussion

The peripheral T cell receptor repertoire is known to be shaped by three main selection mechanisms - thymic positive and negative selection and antigen-driven peripheral selection (30–32). We have here shown that this repertoire is affected by another major selection step occurring in the bone marrow or in the thymus at or before the double-positive stage - a structural mechanism leading to preferred *V_β_* - *J_β_* pairing.

The genetic engineering of T cells for immunotherapy is one of the best hopes for novel cancer treatments (33). Such receptors are optimized to recognize a p-MHC target. However, the results presented here suggest that the target affinity maybe not be the only goal to optimize. To ensure that the resulting receptors are structurally stable, one may favor receptors produced naturally. We have here shown in such receptors one type of bias - preferred *Vβ* - *Jβ* pairing, associated with a preference for intermediate length, molecular weight and polarity of the CDR3. However, other biases may have to be incorporated when developing artificial TCRs.

We have shown that preferred *V_β_* - *J_β_* pairing is ubiquitous and shared in all datasets studied in F clones, with some preferential pairing occurring in NF clones too. We proposed multiple evidences for structural selection events. Specifically, we showed that the frequency of human *V_β_ J_β_* combinations deviates from the one expected based on random pairing and each gene usage frequency. Preferred *V_β_* - *J_β_* pairs are shared between samples and between datasets. This sharing (as measured by the correlation coefficient) is maximal in samples within a donor. The correlations within each host are much higher than the correlations between different hosts, and the correlation between CD4 and CD8 T cell samples is higher than between samples from different compartments. Beyond the general distribution, the *V_β_*, *J_β_* pairs most deviating from random pairing are similar in different datasets and conditions. We have analyzed bulk sequencing, and not single-cell data. Our results are robust in different sets sampled with different methods and different primers. We thus believe they should hold in single-cell data.

Finally, very clear pairing was found already in Double Positive samples using both *J_β_* 1 and *J_β_* 2 *J* genes in mice. The simplest molecular explanation for such a preferential pairing would be that *V_β_* - *J_β_* pairs affect the biochemical properties of the receptor, and that receptors on the extremities of the distribution are selected against, as we have shown here for multiple biochemical properties, such as the length in nucleotides, the molecular weight, the charge and the polarity of the receptor. This is consistent with the relation between the V-J combination used and the CDR3 length distribution, especially for pathogen-specific TCRs (34) and the effect of the mouse compartment on the usage frequency of *V_β_*, *J_β_*, and *V_β_* - *J_β_* heterogeneity (35). This is further consistent with the bias in MHC-constrained systems for CDR3 length and amino acid composition. TCRs with CDR3 longer than 13 amino acids were shown to be disfavored, and positively charged and hydrophobic amino acids in CDR3*β* are limited, and cysteine-containing CDR3 peptide-binding regions are clonally deleted (8).

While *J_β_* usage is conserved between datasets, *V_β_* is not. However, the pairing between *V_β_* and *J_β_* is actually even more conserved than the usage of *V_β_* (**Appendix Figure 9**).

Previous studies have shown *V_β_ J_β_* pairs that are frequent in TCR repertoires. However, to the best of our knowledge, no previous results reported a consistent deviation of *V_β_ J_β_* usage from the one expected randomly.

*V_β_* (*D_β_*) *J_β_* recombination is the main diversity generation mechanism in receptor repertoires. This diversity derives in large part from the multiple combinations of possible joining events and through an inherent imprecision in the joining reaction (36, 37). This large diversity is then reduced step by step by multiple selection events. Our results suggest that beyond the antigen induced selection steps, there is a strong structural selection step. The simplest mechanism would be a preferred length for the CDR3 or a total weight for this CDR3. This could be the result for example of the need to maintain a given surface to bind the MHC, or ensure the curvature of the CDR3, again to ensure proper binding to the HLA.

While we have focused on a specific measure - the *V_β_ J_β_* usage, such a selection may be observed in multiple other measures, such as the CDR3 amino acid usage or the pairing between *α* and *β* chains. Tests should be developed for the detection of such a selection in these other measures, and to estimate the fraction of the TCRs removes following structural selection.

As for the bias in *V_H_* and *J_H_* pairing in B cells, it is more complex based on the structure of *D_H_* in IGH (there are not only two *D_H_* genes as in the T cells, and each *D_H_* can bind each *J_H_*). Thus, a different analysis is required, since we cannot neutralize the effect of *D_H_*. We now explore this as a follow-up work.

## Data Availability Statement

Publicly available datasets were analyzed in this study. This data can be found here: https://elifesciences.org/articles/54747 https://link.springer.com/article/10.1186/s13073-021-00918-7 https://www.nature.com/articles/ng.3822?report=reader https://www.nature.com/articles/s41467-019-08906-7.

## Author Contributions

RL performed the analysis, produced the figures and wrote a part of the manuscript. YL supervised and conceptualized the analysis and wrote a part of the manuscript. All authors contributed to the article and approved the submitted version.

## Funding

The work of RL was funded by ISF grant 870/20 and by an internal BIU DSI grant.

## Conflict of Interest

The authors declare that the research was conducted in the absence of any commercial or financial relationships that could be construed as a potential conflict of interest. t:

## Publisher’s Note

All claims expressed in this article are solely those of the authors and do not necessarily represent those of their affiliated organizations, or those of the publisher, the editors and the reviewers. Any product that may be evaluated in this article, or claim that may be made by its manufacturer, is not guaranteed or endorsed by the publisher.

## Figures and Tables

**Figure 9:**
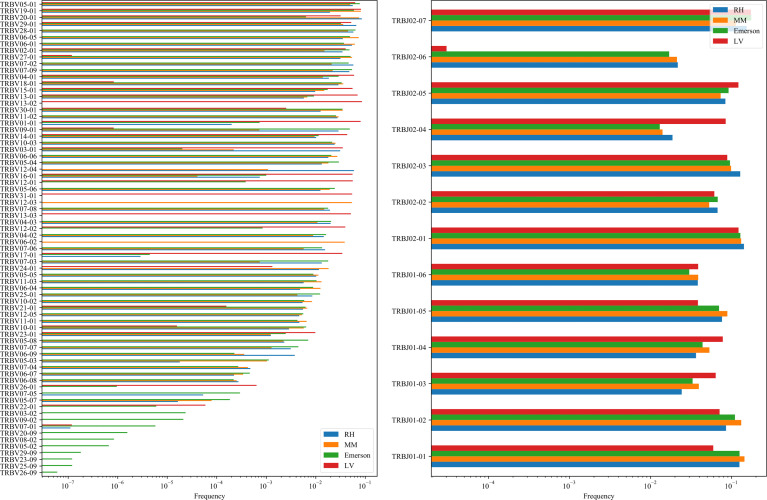
V_β_ **(A)** and J_β_ **(B)** distribution.
